# Effect of mass media on comprehensive knowledge of HIV/AIDS and its spatial distribution among reproductive-age women in Ethiopia: a spatial and multilevel analysis

**DOI:** 10.1186/s12889-020-09536-1

**Published:** 2020-09-17

**Authors:** Chilot Desta Agegnehu, Getayeneh Antehunegn Tesema

**Affiliations:** 1 School of Nursing, College of Medicine and Health Sciences and Comprehensive specialized hospital, University of Gondar, Gondar, Ethiopia; 2 Department of Epidemiology and Biostatistics, Institute of Public Health, College of Medicine and Health Sciences, University of Gondar, Gondar, Ethiopia

**Keywords:** Mass media, Comprehensive knowledge of HIV/AIDS, Multilevel analysis, Spatial analysis

## Abstract

**Background:**

Globally, HIV/AIDS remains a significant public health issue particularly in Sub-Saharan Africa. Media exposure plays a significant role in raising community knowledge about HIV. Therefore, this study aimed to investigate the effect of media on comprehensive knowledge of HIV and its spatial distribution among reproductive-age women in Ethiopia.

**Methods:**

A secondary data analysis was done based on the 2016 Ethiopian Demographic and Health Surveys (EDHS). A total weighted sample of 15,683 reproductive-age women was included for analysis. For the spatial analysis, ArcGIS version 10.3 and SaTScan version 9.6 software were employed to explore the spatial distribution of comprehensive knowledge of HIV/AIDS and for identifying significant hotspot areas. For associated factors, the mixed-effect logistic regression model was fitted. Deviance and ICC were used for model comparison. In the multivariable analysis, Adjusted Odds Ratio (AOR) with 95% Confidence Interval (CI) was reported to declare significantly associated factors of comprehensive knowledge of HIV/AIDS.

**Results:**

The spatial analysis revealed that the spatial distribution of comprehensive knowledge of HIV/AIDS among reproductive-age women was significantly varied across the country. The SaTScan analysis identified significant clusters in the entire Somali region, the eastern part of Dire Dawa and Harari regions. Being rural (AOR = 1.52,95% CI:1.21–1.91), maternal age 25–34 years (AOR = 1.26,95% CI:1.14–1.40), aged ≥35 years (AOR = 1.20,95%CI:1.07–1.35), being Muslim (AOR = 0.68,95% CI:0.60–0.78), being protestant (AOR = 0.83,95% CI:0.71–0.96), poorer wealth (AOR = 1.26,95%CI:1.06–1.51), middle wealth (AOR = 1.34,95%CI:1.11–1.60), richer wealth (AOR = 1.36,95% CI:1.12–1.63), richest wealth (AOR = 1.72,95% CI:1.37–2.15), reading newspaper (AOR = 1.20,95%CI: 1.06–1.37), listening radio (AOR = 1.24,95% CI:1.10, 1.41), covered by health insurance (AOR = 1.23,95%CI:1.01–1.51), having primary education (AOR = 1.77,95% CI:1.57–1.99), having secondary education (AOR = 2.45,95%CI:2.10–2.86) and having higher education (AOR = 3.04,95%CI:2.52–3.65) were significantly associated with comprehensive knowledge of HIV/AIDS.

**Conclusion:**

Spatial distribution of comprehensive knowledge of HIV/AIDS among reproductive-age women was significantly varied across the country with significant hotspot areas with poor comprehensive knowledge of HIV/AIDS identified in the Somali region, the eastern part of Dire Dawa and Harari Region*s. media* exposure was a significant predictor of comprehensive knowledge of HIV/AIDS among reproductive-age women in Ethiopia. Therefore, the government should scale up public health programs in the hot spot areas and provide health information using different media.

## Background

Globally, particularly in Sub-Saharan Africa (SSA), Human Immunodeficiency Virus /Acquired Immune Deficiency Syndrome (HIV/AIDS) was the leading cause of morbidity and mortality among reproductive-age women [1]. In sub-Saharan Africa, it is estimated that 60% of HIV/AIDS patients were reproductive-age women [2] however, less than 30% of reproductive-age women have comprehensive knowledge of HIV/AIDS [3]. In developing countries like Ethiopia, reproductive-age women are highly infected by HIV/AIDS owing to the discriminatory cultural, social, and economic status in society [4]. Mass media exposure (television, radio, and newspapers) have become a significant source of raising awareness on HIV/AIDS to the population and an effective tool for disseminating HIV education to the group [5, 6]. Media exposure is one of the most common and cost-effective public health programs globally for improving public health issues by increasing knowledge and altering health behaviors [7]. The level of HIV/AIDS-related knowledge among reproductive-age women has varied across and within countries and strongly connected to the socio-economic contexts [8].

In the last few years, SSA countries have presented media exposure to combat HIV/AIDS to rapidly touch the community by addressing different messages [9]. However, mass media availability and accessibility have been speckled across different social groups [10, 11], and it is the most powerful tool to change the health behavior of the community [12]. To reduce the incidence of HIV/AIDS among reproductive-age women, improving comprehensive knowledge of HIV/AIDS transmission, and smearing ways of prevention methods is a crucial issue [13].

Despite mass media plays a significant role in reducing the incidence of HIV/AIDS through raising comprehensive knowledge towards HIV/AIDS, most of the reproductive-age women in developing countries including Ethiopia have poor comprehensive knowledge of HIV/AIDS. For example, in Sub-Saharan Africa Uganda, only 38% of reproductive-age women had a comprehensive knowledge of HIV/AIDS [14]. Besides, in Ethiopia especially in Afar, Gambella, Dire Dawa, and Somali regions were detected poor knowledge of HIV/AIDS among reproductive-age women [15]. However, there are few studies conducted on the magnitude and determinants of comprehensive knowledge of HIV/AIDS among reproductive-age women in Ethiopia [16, 17], these studies are unable to capture the spatial distribution of comprehensive knowledge of HIV/AIDS and the effect of media exposure on the comprehensive knowledge of HIV/AIDS. Therefore, this study aimed to investigate the effect of mass media on comprehensive knowledge of HIV/AIDS and its spatial distribution among reproductive-age women in Ethiopia using spatial and multilevel analysis. Thus, the findings of this study could help to increase media exposure in the identified hotspot areas where poor comprehensive knowledge of HIV/AIDS was clustered to reduce the incidence of HIV/AIDS among reproductive-age women in Ethiopia.

### Study design, setting, and period

A community-based cross-sectional study was conducted based on the 2016 Ethiopian Demographic and Health Survey (EDHS) data. It was the fourth nationally representative survey conducted in Ethiopia employed with a 5-year interval. Ethiopia is situated in the Horn of Africa. It has 9 Regional states (Afar, Amhara, Benishangul-Gumuz, Gambela, Harari, Oromia, Somali, Southern Nations, Nationalities, and People’s Region (SNNP) and Tigray) and two Administrative Cities (Addis Ababa and Dire-Dawa). In Ethiopia, about 84% of the population are rural residents [18]. It is the13^th^ in the world and 2nd in Africa’s most populous country [19].

### Sample and source of population

All reproductive-age women within 5 years preceding the survey in Ethiopia were the source of the population whereas all reproductive-age women in the selected enumeration areas within 5 years preceding the survey were the study population. In EDHS, a stratified two-stage cluster sampling technique was employed using the 2007 Population and Housing Census (PHC) as a sampling frame. In the first stage, 645 Enumeration Areas (EAs) were selected. In the second stage, on average 28 households were systematically selected. A total weighted sample of 15,683 reproductive-age women was included in the study. The detailed sampling procedure is presented in the full EDHS 2016 report [20].

### Measurement of variables

The dependent variables used for the study was **“**comprehensive knowledge of HIV/AIDS”, it was generated by aggregating a series of questions which were designed to evaluate knowledge of HIV/AIDS was based on the question: 1**)** the risk of HIV/AIDS transmission can be reduced by having sex with only one infected partner, who has no other partners, 2) a person can reduce the risk of getting HIV by using a condom every time they have sex; 3) a healthy-looking person can have HIV; 4) a person can get HIV from mosquito bites; 5) a person can get HIV by sharing food with someone infected. These questions were used by Millennium Developing Goals (MDG) to measure “comprehensive knowledge of HIV/AIDS” in this study. Then the outcome variable was coded as 0 = “No” if women didn’t have comprehensive knowledge of HIV/AIDS, and as 1 = “yes” if a woman had Comprehensive knowledge of HIV/AIDS.

Independent variables included in this study were frequency of media exposure (frequency of watching television, listening radio, and reading newspaper, which was defined as “not at all”, “less than once a week” and “at least once a week”, maternal age, residence, place of delivery, covered by health insurance, occupation, maternal age, religion, wealth status, and maternal education.

### Data management and analysis

STATA version 14, ArcGIS version 10.6, and SaTScan version 9.6 statistical software were used for analysis. The DHS data was hierarchal data, there might have a clustering effect hence women in one cluster might share similar characteristics than women in different clusters. Therefore we have checked the clustering effect using the Intra-class Correlation Coefficient (ICC) and Likelihood Ratio (LR) test. The ICC indicates that there was a significant clustering effect that should be considered using advanced models such as mixed-effect models. We have fitted two models such as the standard logistic regression model and the mixed-effect logistic regression model. Deviance, Akakie Information Criteria (AIC), and Bayesian Information Criteria (BIC) were used for model comparison. The mixed-effect logistic regression model was the best-fitted model for the data since it had the lowest deviance value. Both bi-variable and multivariable mixed-effect logistic regression analyses were done. Variables with < 0.2 *p*-values in the bi-variable analysis were considered for the multivariable mixed-effect logistic regression model. Adjusted Odds Ratio (AOR) with a 95% Confidence Interval (CI) and p-value < 0.05 in the multivariable model were used to declare significant association with comprehensive knowledge of HIV/AIDS. For the determinant factors, we used STATA version 14 statistical software using xtmelogit, xtmrho and icc packages were used.

### Spatial analysis

For the spatial analysis ArcGIS version, 10.3 and SaTScan version 9.6 software were used.

### Spatial autocorrelation analysis

The spatial autocorrelation (Global Moran’s I) analysis is a spatial statistics used to measure spatial autocorrelation by taking the entire data set and produce a single output value which ranges from − 1 to + 1. Moran’s I assess whether the spatial distribution of comprehensive knowledge of HIV/AIDS was dispersed, clustered, or randomly distributed in the study area [21]. Moran’s I values close to − 1 indicates there is dispersion, whereas Moran’s I close to + 1 indicate there is spatial clustering and distributed randomly if Moran’s I value is close to 0.

### Hot spot analysis (Getis-OrdGi* statistic)

Getis-OrdGi* statistics were computed to measure how spatial autocorrelation varies over the study location by calculating GI* statistic for each area. Z-score is computed to determine the significant hotspot and significant cold spot areas of poor comprehensive knowledge towards HIV/AIDS. Statistical output with high GI* indicates “hotspot” whereas low GI* indicates “cold spot” [22].

### Spatial interpolation

The Kriging spatial interpolation technique was used to predict the percentage of comprehensive knowledge towards HIV/AIDS among reproductive-age women on the un-sampled areas in the country based on observed measurements. There are various deterministic and geostatistical interpolation methods. Ordinary Kriging spatial interpolation method was used for predictions of comprehensive knowledge of HIV/AIDS in unobserved areas of Ethiopia since it incorporates the spatial autocorrelation and it statistically optimizes the weight [23].

### Spatial scan statistical analysis

For the spatial scan statistical analysis, the Bernoulli based model was employed to test for the presence of statistically significant spatial clusters of poor comprehensive knowledge of HIV/AIDS using Kuldorff’s SaTScan version 9.6 software. The SaTScan uses a circular scanning window that moves across the study area. Women having poor comprehensive knowledge of HIV/AIDS were taken as cases and those who have good comprehensive knowledge of HIV/AIDS as controls to fit the Bernoulli model. The numbers of cases in each location had Bernoulli distribution and the model required data for cases, controls, and geographic coordinates. The default maximum spatial cluster size of < 50% of the population was used, as an upper limit, which allowed both small and large clusters to be detected and ignored clusters that contained more than the maximum limit.

For each potential cluster, a likelihood ratio test statistic and the *p*-value were used to determine if the number of observed comprehensive knowledge of HIV/AIDS within the potential cluster was significantly higher than expected or not. The scanning window with maximum likelihood was the most likely performing cluster, and the *p*-value was assigned to each cluster using Monte Carlo hypothesis testing by comparing the rank of the maximum likelihood from the real data with the maximum likelihood from the random datasets. The primary and secondary clusters were identified and assigned *p*-values and ranked based on their likelihood ratio test, based on 999 Monte Carlo replications [24].

## Results

### Descriptive characteristics of comprehensive knowledge of HIV/AIDS

A total of 15,683 reproductive-age women were included in the study, of these, 94.6% of the women in the Somali region had a poor comprehensive knowledge of HIV/AIDS, whereas nearly half (50.2%) of the reproductive age women in Addis Ababa have good comprehensive knowledge of HIV/AIDS. According to the residence, 80.6% of reproductive age women residing in a rural area had a poor comprehensive knowledge of HIV/AIDS. The majority (57.5%) of the women who attained a higher level of education had a good comprehensive knowledge of HIV/AIDS. Besides, more than three-fourth (84.2. %) of the women from the poorest household wealth had a poor comprehensive knowledge of HIV/AIDS (Table 1).

**Table 1:** Background characteristics of respondents (*N* = 15,683)

Variable	Comprehensive knowledge of HIV/AIDS	
	Yes (%)	No (%)
**Region**		
Tigray	31.2	68.8
Afar	14.7	85.4
Amhara	27.3	72.7
Oromia	23.1	76.9
Somali	5.3	94.6
Benishangul	17.6	82.4
SNNPR	19.0	81.0
Gambella	25.6	74.4
Harari	22.8	77.2
Addis Ababa	50.5	49.5
Diredawa	23.9	76.1
**Residence**		
Rural	19.4	80.6
Urban	43.8	56.2
**Maternal age**		
15–24	27.1	72.9
25–34	24.6	75.4
≥ 35	21.7	78.3
**Respondent working**		
No	21.6	78.4
Yes	28.0	72.0
**Religion**		
Orthodox	30.3	69.7
Muslim	20.4	79.6
Catholic	7.7	92.3
Protestant	21.7	78.3
Traditional	15.3	84.7
**Wealth status**		
Poorest	15.8	84.2
Poorer	18.1	81.9
Middle	19.2	80.8
Richer	21.8	78.2
Richest	41.3	58.7
**Maternal Education**		
No education	16.0	84.0
Primary education	25.7	74.3
Secondary education	42.6	57.4
Higher	57.5	42.5
**Place of delivery**		
Home	24.1	75.9
Health facility	28.4	71.6
**Covered by health insurance**		
No	24.5	75.5
Yes	29.7	70.3

### Media exposure status of respondents

Of the total 15,683 women, 10,485 (66.9%) have never listened to the radio and about 2581 (16.4%) had listened to the radio at least once a week. Regarding television, about 11,294 (72.0%) never watching television, and 2485 (15.9%) were watching television at least once a week (Table 2).

**Table 2:** The distribution of respondent’s exposure to mass media

Variables	Frequency (%)
**Frequency of listening radio**	
Not at all	10,485 (66.9)
Less than once a week	2617 (16.7)
At least once a week	2581 (16.4)
**Frequency of watching television**	
Not at all	11,294 (72.0)
Less than once a week	1904 (12.1)
At least once a week	2485 (15.9)
**Frequency of reading newspaper/ magazines**	
Not at all	13,548 (86.4)
Less than once a week	1516 (9.7)
At least once a week	619 (3.9)

### The proportion of comprehensive knowledge of HIV/AIDS

**Fig. 1:**
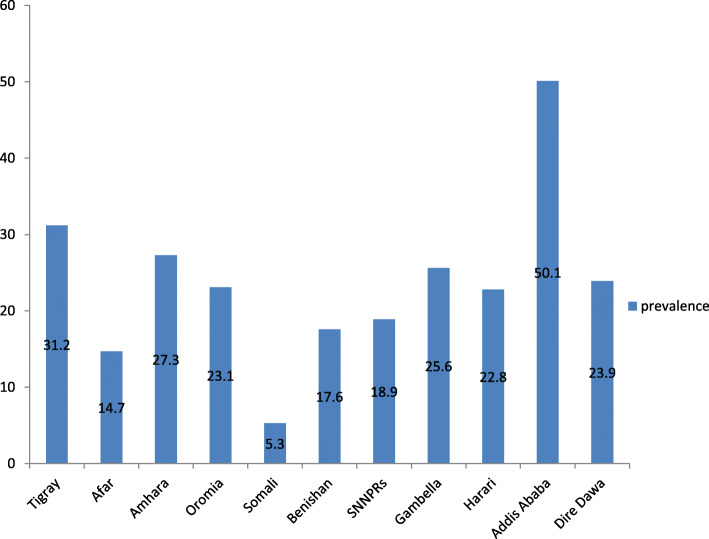
The prevalence of comprehensive knowledge of HIV/AIDS among reproductive-age women in Ethiopia 2016 EDHS

The proportion of good comprehensive knowledge of HIV/AIDS among reproductive-age women in Ethiopia was 24.8% (95% CI, 24.1, 25.5%) ranging from 5.3% in the Somali region to 50.5% in Addis Ababa (Fig. 1).

### Spatial analysis

#### Spatial distribution of comprehensive knowledge of HIV/AIDS

**Fig. 2:**
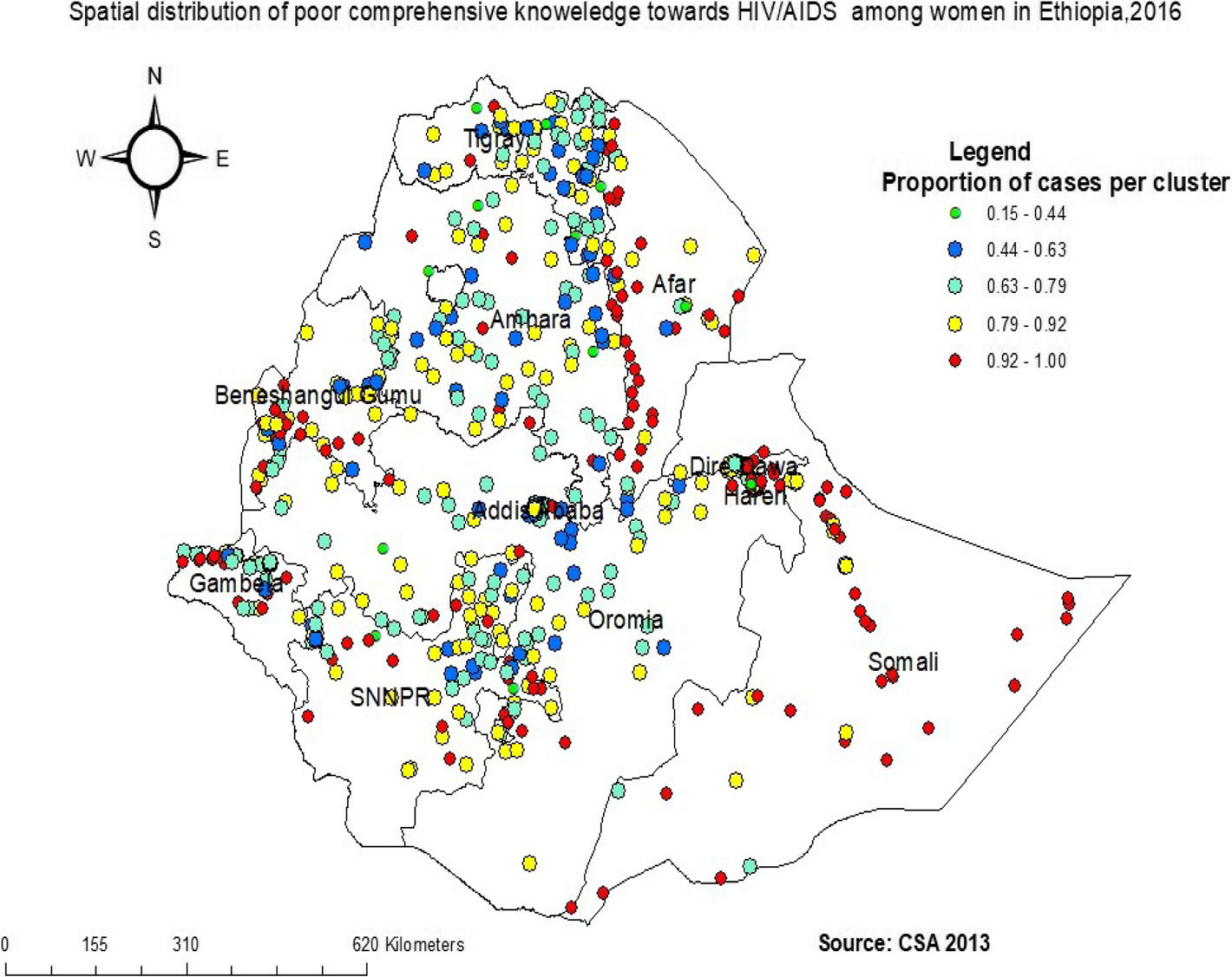
Spatial distribution of Comprehensive knowledge of HIV/AIDS among reproductive-age women in Ethiopia, 2016

A total of 622 clusters were included in the spatial analysis of comprehensive knowledge of HIV/AIDS. The spatial distribution of comprehensive knowledge of HIV/AIDS among reproductive age was significantly varied across the country with Global Moran’s Index 0.33 (*p* < 0.001). The high proportion of poor comprehensive knowledge of HIV/AIDS (the red color) was aggregated in the northeast and southwest Somali, Dire Dawa and Harari, south and northwest part of Afar, West Gambela, Benishangul Gumuz, and East and North part of SNNP regions. Whereas the low proportion of poor comprehensive knowledge of HIV/AIDS (yellow color) has occurred in central Tigray, Addis Ababa, and North-East part of Amhara regions (Fig. 2).

#### Hot spot analysis of comprehensive knowledge of HIV/AIDS

**Fig. 3:**
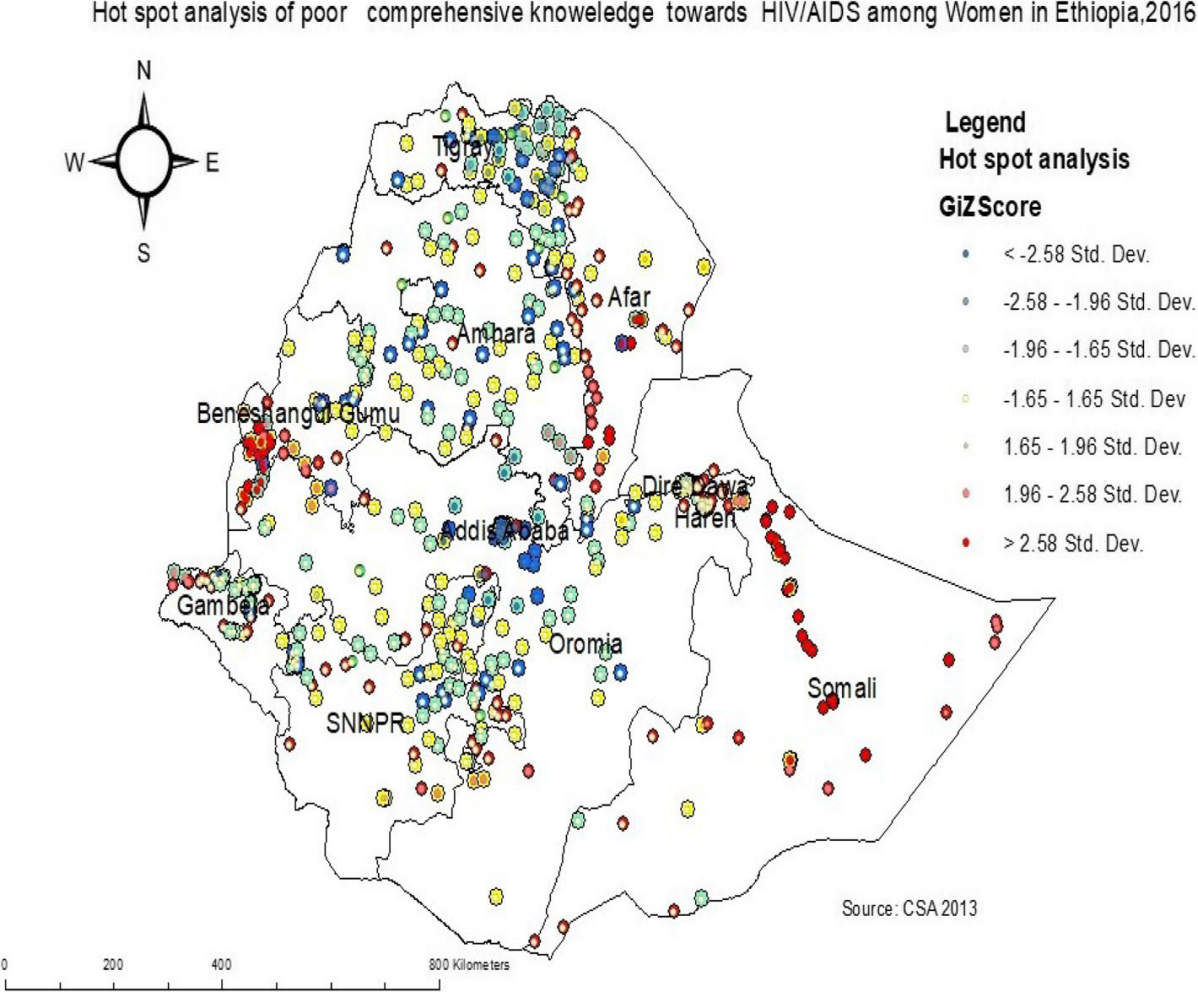
Hot spot and cold spot identifications of comprehensive knowledge of HIV/AIDS among reproductive-age women in Ethiopia, 2016

In the hot spot analysis, the significant hot spot areas of poor comprehensive knowledge of HIV/AIDS were found in northeast Somali, southwest Afar, southwest Benishangul Gumuz, and northwest Gambela regions (*P* < 0.01). Whereas the significant cold spot areas of poor comprehensive knowledge of HIV/AIDS were identified in central Tigray, Addis Ababa, northeast Amhara, and east SNNP regions (Fig. 3).

#### Interpolation of poor comprehensive knowledge of HIV/AIDS

**Fig. 4:**
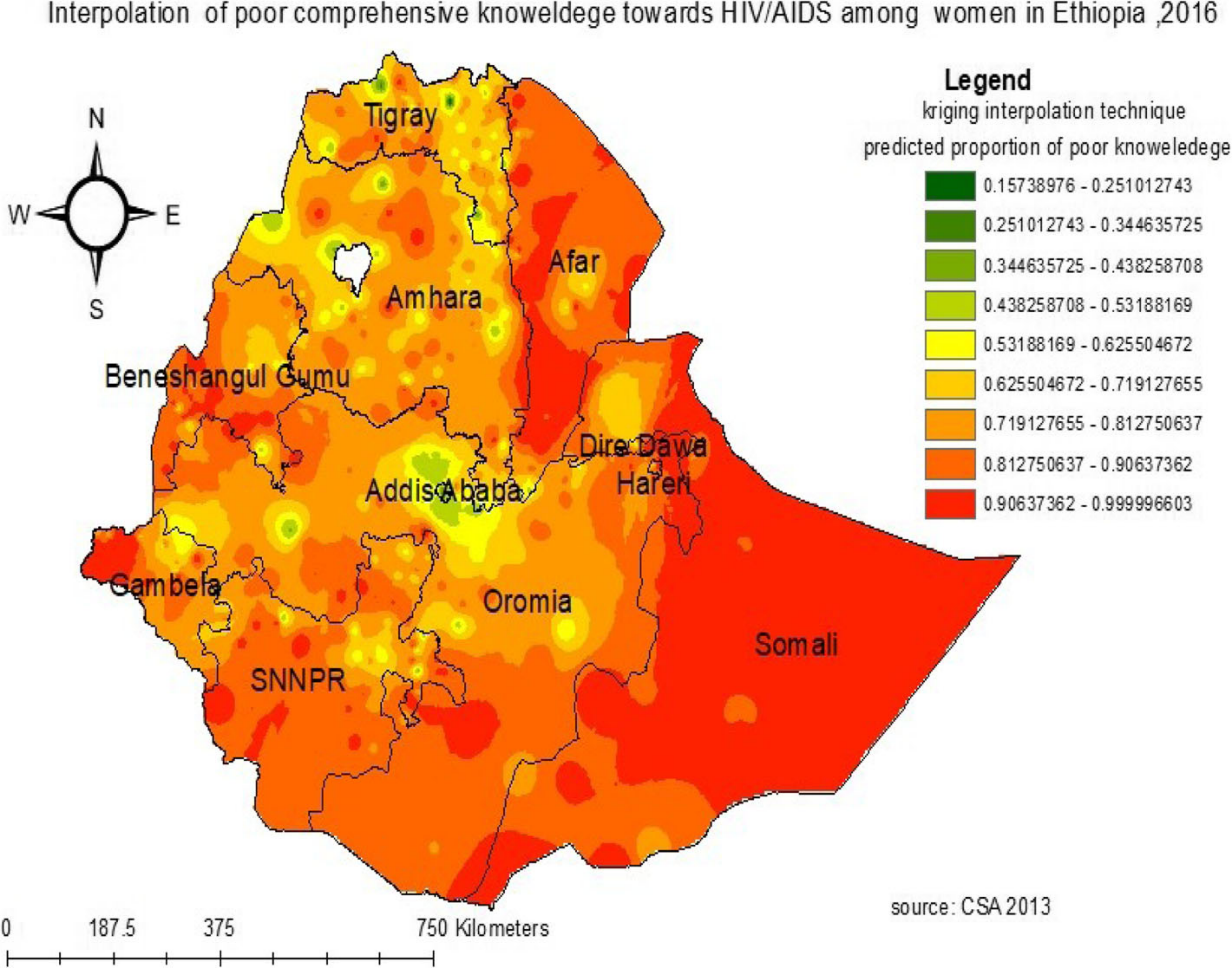
kriging interpolation of poor comprehensive knowledge of HIV/AIDS among reproductive-age women in Ethiopia, 2016

Based on Kriging interpolation, the predicted high-risk areas of poor comprehensive knowledge of HIV/AIDS among reproductive-age women were identified in Somali, southwest Afar, and west Gambela regions. Whereas the eastern part of Addis Ababa and north Tigray regions were identified as predicted low risky areas of poor comprehensive knowledge of HIV/AIDS (Fig. 4).

#### Spatial sat scan analysis of poor comprehensive knowledge HIV/AIDS

**Fig. 5:**
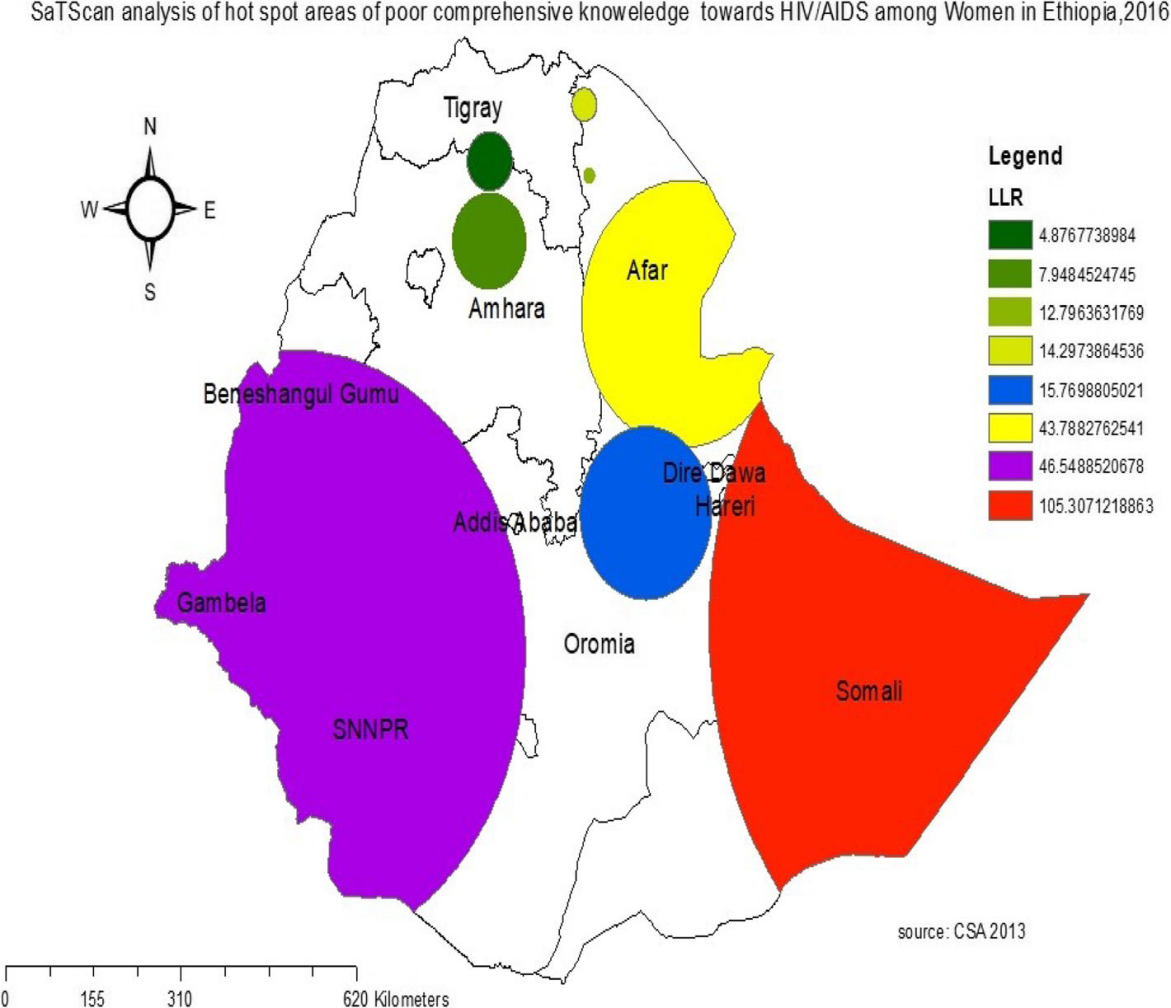
Sat scan analysis of poor comprehensive knowledge of HIV/AIDS among reproductive-age women in Ethiopia, 2016. We would like to acknowledge the Central Statistics Agency 2013 for the source of shapefiles. The map was generated by using the software Arc GIS 10.3

A total of 312 significant primary and secondary clusters of poor comprehensive knowledge of HIV/AIDS were identified. The primary clusters were located in the entire Somali, the eastern part of Dire Dawa, and Harari regions centered at 7.650693 N, 47.007920 E with a radius of 565.01 km, a relative risk (RR) of 1.28, and the Log-Likelihood Ratio (LLR) of 105.3, at *P*-value< 0.001. This showed that reproductive-age women within the spatial window had 1.28 times more likely to have poor comprehensive knowledge of HIV/AIDS as compared to reproductive age women outside the spatial window (Fig. 5) (supplementary file 1).

#### Determinants of comprehensive knowledge of HIV/AIDS

Model comparison: AIC, BIC, and deviance (−2LL) were used for model comparison, and the mixed effect logistic regression model was the best-fitted model because of the smallest value of deviance (Table 3). Furthermore, the ICC value was 0.17 (95% CI: 0.16, 0.20) and the Log-likelihood ratio test was (chibar2 < 0.001) which informed us of the mixed effect logistic regression model (GLMM) the best-fitted model over the basic model.

**Table 3:** Model comparison

Model comparison	AIC	BIC	Deviance
Logistic regression model **Mixed effect logistic regression model**	15,493.95 **14,981.77**	15,677.8 15,173.28	15,445.95 **14,931.772**

In the multivariable mixed-effect logistic regression; maternal education, maternal age, wealth status, occupational status, media exposure, residence, religion, and health issuance coverage were significantly associated with the comprehensive knowledge of HIV/AIDS.

Women residing in the rural area were 1.52 (AOR = 1.52, 95% CI: 1.21, 1.91) times higher odds of having good comprehensive knowledge of HIV/AIDS compared to urban women. The odds of having good comprehensive knowledge of HIV/AIDS among women aged 25–34 years and ≥ 35 years were 1.26 (AOR = 1.26, 95%CI: 1.14, 1.40) and 1.20 (AOR = 1.20, 95%CI: 1.07, 1.35) times higher than women aged 15–24 years respectively. The odds of having comprehensive knowledge of HIV/AIDS among Muslim and protestant religious followers were decreased by 32% (AOR = 0.68, 95% CI: 0.60, 0.70) and 17% (AOR = 0.83, 95% CI: 0.71, 0.96) than orthodox Christian followers respectively. Women in the poorer, middle, richer, and richest household wealth were 1.26 (AOR = 1.26, 95%CI: 1.06, 1.51), 1.34 (AOR = 1.34, 95%CI: 1.11, 1.60), 1.36 (AOR = 1.36, 95%CI: 1.12, 1.63), and 1.72 (AOR = 1.71, 95%CI: 1.37, 2.15) times higher odds of having good comprehensive knowledge of HIV/AIDS respectively compared to women in the poorest household wealth. Women who attained primary education, secondary, and higher education were 1.77 times (AOR = 1.77, 95% CI: 1.47, 1.99), 2.45 times (AOR = 2.45, 95%CI: 2.10, 2.86), and 3.04 times (AOR = 3.04, 95%CI: 2.52, 3.63) higher odds of having good comprehensive knowledge of HIVAIDS compared to women didn’t attain education respectively. Women who were covered by health insurance were 1.23 (AOR = 1.23, 95%CI: 1.01, 1.51) times higher odds of having good comprehensive knowledge of HIV/AIDS.

Those who read newspaper or magazines less than once a week (AOR = 1.20, 95%CI: 1.06, 1.37), who listened to the radio at least once a week (AOR = 1.24, 95%CI: 1.10, 1.41), and who watched television at least once a week (AOR = 1.32, 95%CI: 1.13, 1.54) had higher odds of having good comprehensive knowledge of HIV/AIDS (Table 4).

**Table 4:** Bi-variable and Multivariable mixed-effect logistic regression analysis of determinants of comprehensive knowledge of HIV/AIDS among reproductive-age women in Ethiopia from January 18 to June 27, 2016

Variables	COR (95% CI)	AOR (95% CI)
**Residence**		
Urban	4.45 [3.70, 5.34]	1.52 [1.21, 1.91]**
Rural	1	1
**Maternal age**		
15–24	1	1
25–34	1.06 [0.96, 1.16]	1.26 [1.14, 1.40]**
≥ 35	0.86 [0.78, 0.96]	1.20 [1.07, 1.35]**
**Religion**		
Orthodox Christian	1	1
Muslim	0.52 [0.45, 0.60]	0.68 [0.60, 0.78]**
Protestant	0.77 [0.65, 0.90]	0.83 [0.71, 0.96]**
Catholic	0.57 [0.31, 1.06]	0.55 [0.29, 1.03]
Traditional/other	0.72 [0.42, 1.21]	0.98 [0.59, 1.64]
**Working status**		
Working	1.23 [1.13, 1.35]	1.13 [1.03, 1.24]**
Not working	1	1
**Wealth status**		
Poorest	1	1
Poorer	1.42 [1.19, 1.70]	1.26 [1.06, 1.51]**
Middle	1.65 [1.37, 1.98]	1.34 [1.11, 1.60]**
Richer	1.93 [1.60, 2.32]	1.36 [1.12, 1.63]**
Richest	4.46 [3.74, 5.33]	1.72 [1.37, 2.15]**
**Place of delivery**		
Home	1	1
Health facility	1.12 [1.01, 1.24]	1.02 [0.92, 1.14]
**Reading newspaper/ magazines**		
Not at all	1	1
Less than once a week	1.91 [1.70, 2.15]	1.20 [1.06, 1.37]**
At least once a week	1.86 [1.56, 2.23]	1.05 [0.87, 1.27]
**Frequency of listening radio**		
Not at all	1	1
Less than once a week	1.45 [1.29, 1.62]	1.10 [0.98, 1.25]
At least once a week	1.83 [1.63, 2.05]	1.24 [1.10, 1.41]**
**Frequency of watching TV**		
Not at all	1	1
Less than once a week	1.63 [1.42, 1.88]	1.01 [0.87, 1.18]
At least once a week	2.76 [2.42, 3.15]	1.32 [1.13, 1.54]**
**Covered by health insurance**		
No	1	1
Yes	1.37 [1.11, 1.68]	1.23 [1.01, 1.51]**
**Maternal education**		
No education	1	1
Primary education	1.93 [1.73, 2.14]	1.77 [1.57, 1.99]**
Secondary education	3.17 [2.77, 3.62]	2.45 [2.10, 2.86]**
Higher education	4.66 [3.96, 5.49]	3.04 [2.52, 3.65]**

*p-value< 0.05 significantly associated

## Discussion

This study aimed to investigate the effect of media exposure on comprehensive knowledge of HIV/AIDS and its spatial distribution. This study is the first in Ethiopia that explore the spatial distribution of comprehensive knowledge of HIV/AIDS in the country. This study has significant public health implications for designing effective public health interventions to reduce the incidence of HIV/AIDS at the national level.

In this study, the proportion of good comprehensive knowledge of HIV/AIDS among reproductive-age women in Ethiopia was 24.8%. There was significant spatial variation in the spatial distribution of comprehensive knowledge of HIV/AIDS across the country, the significant hotspot areas of poor comprehensive knowledge were identified in the northwest Somali, southwest Afar, southwest Benishangul Gumuz, and northwest Gambela regions. Besides the primary significant clusters were located in Somali, the eastern part of Dire Dawa and Harari regions. The possible justification could be due to the difference in the distribution of maternal health care services across regions, hence the border areas of the country are facing health care inaccessibility and inaccessibility of infrastructure in the border areas of Somali, and Benishangul regions [25], therefore, women in this area missed health education about HIV/AIDS in the health facilities this might be the possible explanation for the poor comprehensive knowledge of HIV/AIDS in this areas. Besides, these areas are more of rural residents and had poor access to mass media this could be contributed to the poor comprehensive knowledge of HIV/AIDS among reproductive-age women. Women’s education plays a great role in their economic, socio-cultural, and political empowerment [26]. Furthermore, these regions are highly marginalized and the community had poor access to education, and commonly the husband had the power to decide on the women’s health [27]. Furthermore, evidence documented that in the Somali region there is limited maternal health care services accessibility a utilization [28, 29].

In the multivariable mixed-effect logistic regression analysis; residence, religion, listening to the radio, watching television, reading the newspaper, health insurance, maternal education, occupation status, maternal age, and wealth status were significantly associated with good comprehensive knowledge of HIV/AIDS among reproductive-age women.

In this study, media exposure was significantly associated with comprehensive knowledge of HIV/AIDS among reproductive-age women. Women who had media exposure had higher odds of having good comprehensive knowledge of HIV/AIDS compared to women who didn’t have media access. It is in line with studies reported in China [30], Ghana [31], Nigeria [32], and Uganda [33], this could be because mass media (radio, television, and newspaper) are the major source of information, and most powerful for addressing a large group of people to change the community awareness, attitude and practice towards HIV/AIDS [34] this could be the possible reason. Mass communication such as television, radio, and newspaper increases the sexual health knowledge of the population through delivering a repeated message about their methods of transmission and mode of transmission of HIV/AIDS and warning about the risk and complications of HIV/AIDS. The importance of mass media in health promotion and disease prevention is well documented, since both routine exposure to and strategic use of mass media play a significant role in promoting awareness, increasing knowledge, and changing health behaviors.

Women residing in urban areas had higher odds of having good comprehensive knowledge of HIV/AIDS than rural residents. This was supported by the previous study findings in South Africa [35] and Canada [36], the possible explanation might be due to urban residents had access to media exposure and health promotion services relatively than that of rural residents [37].

Women from poor household wealth had lower odds of good comprehensive knowledge of HIV/AIDS as compared to women from rich household wealth. This was in line with studies done in Uganda [38]. This could be due to the reason that women from the richest household had a high wealth status had a better standard of living and can easily afford and access health information from social media and other social services [39].

Women covered by health insurance had higher odds of having good comprehensive knowledge of HIV/AIDS as compared to those who were not covered by health insurance. It was supported by research is done in Vietnam [40] since health insurance plays an important role in ensuring health equity and the aim of health insurance is to make counseling service, medical and reproductive health service accessible to the community [41]. Therefore, health insurance can increase the community’s health care service utilization [42], this could raise the awareness of the community towards HIV/AIDS.

Muslim and protestant religious followers had lower odds of having good comprehensive knowledge of HIV/AIDS as compared to Orthodox Christians’ religious followers. This could be due to the misconceptions followers towards HIV/AIDS prevention and their route of transmission as well as the deeply rooted cultural beliefs towards HIVAIDS of religion [43, 44].

Educated women had higher odds of having comprehensive knowledge of HIV. There are similar studies in Bangladesh [45] and Zimbabwe [46], it suggests that the information disseminated to promote the community has not been clearly understood equally by women who are not educated [47]. These suggest that we have to provide information in a manner that is understandable by all groups of women.

Women who had occupations had higher odds of good comprehensive knowledge of HIV/AIDS as compared to those who don’t have the occupation. This was supported by a study reported in Zimbabwe [46], and it suggests that work enables women to have a social link with people who have knowledge of HIV and they might have sharing of ideas and learning each other about health promotion activities in the working environment. Consistent with a study done in Vietnam [40], and Bangladesh [45], the likelihood of having comprehensive knowledge of HIV among 15–24 years were lower than women aged greater than 24 years. These show that younger women are less concentrated to absorb information, this could reduce their likelihood of knowing about HIV/AIDS and older women had exposure to health promotion services.

### Strength and limitation of the study

The strength of the study was based on a weighted nationally representative large dataset and was based on the advanced model to take into account the hierarchical structure of the data. Besides, we have done the spatial analysis and identified significant hotspot areas where comprehensive knowledge of HIV/AIDS is poor. Limitations, as the study was a cross-sectional survey, a temporal relationship cannot be inferred. Furthermore, the SaTScan analysis unable to detect irregularly shaped spatial clusters.

The findings of this study would contribute significantly to design effective public health interventions in increasing comprehensive knowledge of HIV/AIDS through the use of mass media in the identified significant hotspot areas of poor comprehensive knowledge of HIV/AIDS to reduce the incidence of HIV/AIDS at the national level. Furthermore, the spatial analysis result plays a significant role in making priorities to implement public health programs.

## Conclusions

The spatial analysis showed that the spatial distributions of comprehensive knowledge HIV/AIDS were significantly varied across the country. North East Somali, South West Afar, South West Benishangul Gumuz, and North West Gambela region were identified as significant hotspot areas where poor comprehensive knowledge of reproductive age women towards HIV/AIDS was highly clustered. This study found that media exposure was a significant predictor’s comprehensive knowledge of reproductive age women towards HIV/AIDS. Additionally, maternal education, age, religion, residence, media exposure, health insurance, working status, and wealth status were significantly associated with comprehensive knowledge of HIV/AIDS. Therefore, public health interventions targeting rural residents, non-educated women, poorest, and marginalized populations to improve awareness about HIV/AIDS are needed to improve their knowledge towards HIV/AIDS as well as to reduce the incidence of HIV/AIDS.

## Supplementary information

**Additional file 1: Supplementary file 1.** Sat Scan analysis of poor comprehensive knowledge HIV/AIDS among reproductive-age women in Ethiopia, 2016.

## Data Availability

All relevant data are publically available and here is the link of the Ethiopian Demographic and Health Survey 2016 data and you can access the data by clicking the following link https://dhsprogram.com/data/dataset/Ethiopia_Standard-DHS_2016.cfm?flag=0 .

## Abbreviations

AIC: Akaike information criteria
AIDS: Acquired Immune Deficiency Virus
AOR: Adjusted Odds Ratio
BIC: Bayesian information criteria
COR: Crude Odds Ratio
EDHS: Ethiopian Demographic health survey
HIV: Humane Immune Virus
ICC: Intra-Class Correlation
SSA: Sub Saharan Africa
