# Supplementary material for: Effect of mass media on comprehensive knowledge of HIV/AIDS and its spatial distribution among reproductive-age women in Ethiopia: a spatial and multilevel analysis
Source: BMC Public Health. 2020 Sep 17;20:1420. doi: 10.1186/s12889-020-09536-1 (PMC7499843; doi:10.1186/s12889-020-09536-1)
Supplement: Supplementary file 1 — Additional file 1: Supplementary file 1. Sat Scan analysis of poor comprehensive knowledge HIV/AIDS among reproductive-age women in Ethiopia, 2016. [file 12889_2020_9536_MOESM1_ESM.docx]

**Supplementary file1:** Sat Scan analysis of poor comprehensive knowledge HIV/AIDS among reproductive-age women in Ethiopia, 2016

| Cluster | Enumeration area(cluster)identified | Coordinate/radius | Population | Case | RR | LLR | p-value |
| --- | --- | --- | --- | --- | --- | --- | --- |
| 1(53) | 78, 630, 269, 629, 77, 146, 92, 490, 543, 492, 171, 198, 95, 497, 458, 588, 553, 138, 521, 214, 573, 33, 251, 239, 116, 85, 358, 164, 22, 527, 568, 277, 439, 64, 278, 57, 210, 8, 186, 566, 1, 318, 622, 436, 212, 187, 454, 501, 68, 357, 483, 419, 513 | (7.650693 N, 47.007920 E) / 565.01 km | 790 | 737 | 1.28 | 105.3 | <0.001 |
| 2 (197) | 46, 554, 299, 526, 243, 459, 465, 552, 168, 197, 371, 326, 119, 437,177, 325, 477, 376, 448, 446, 219, 555, 586, 270, 207, 593, 265, 154, 284, 114, 231, 489, 469, 47, 221, 291, 417, 549, 63, 106, 558, 13,337, 105, 343, 567, 76, 338, 315, 603, 411, 346, 432, 470, 62, 233,  426, 69, 486, 248, 447, 260, 104, 175, 592, 507, 370, 227, 306, 304, 536, 435, 309, 643, 406, 193, 113, 349, 618, 462, 275, 70, 266, 502,141, 374, 126, 395, 294, 161, 434, 142, 621, 565, 450, 17, 466, 331,180, 87, 124, 577, 280, 41, 360, 88, 174, 86, 416, 505, 53, 335, 272,20, 262, 388, 6, 320, 162, 65, 433, 118, 234, 373, 223, 503, 165, 537, 399, 563, 317, 203, 342, 595, 347, 297, 271, 569, 209, 581, 508,407, 359, 420, 23, 609, 409, 204, 324, 50, 184, 485, 408, 148, 14,215, 633, 216, 244, 32, 574, 634, 308, 183, 285, 494, 150, 182, 139, 364, 137, 457, 445, 578, 232, 517, 35, 600, 54, 391, 12, 36, 217, 21, 365, 313, 559, 576, 522, 589, 246, 468, 533, 316, 398, 405, 218 | **(**7.396504 N, 35.260203 E) / 405.30 km) | 4332 | 3425 | 1.11 | 46.55 | <0.001 |
| 3(30) | 96, 440, 632, 75, 178, 499, 205, 4, 334, 570, 427, 366, 348, 547, 368, 55, 276, 389, 191, 571, 544, 241, 254, 599, 37, 620, 189, 135,344 | (11.494107 N, 41.493043 E) / 179.34 km | 552 | 494 | 1.22 | 43.78 | <0.001 |
| 4(25) | 12, 506, 333, 476, 491, 372, 93, 122, 51, 49, 71, 564, 245, 230, 529, 39, 453, 441, 557, 336, 594, 25, 484, 30 | (9.090824 N, 40.873894 E) / 116.33 km | 483 | 407 | 1.15 | 15.76 | <0.001 |
| 5(4) | 263, 362, 134, 127 | (14.034142 N, 39.898488 E) / 22.09 km | 94 | 89 | 1.29 | 14.29 | <0.001 |
| 6(3) | 130, 511, 172 | 13.169308 N, 39.987117 E) / 10.69 km | 55 | 54 | 1.33 | 12.79 | 0.0017 |
